# Molecular Duality of OGG1: From Genomic Guardian to Redox-Sensitive Modulator in Diseases

**DOI:** 10.3390/antiox14080980

**Published:** 2025-08-10

**Authors:** Ranwei Zhong, Weiran Zhang, Xiangping Qu, Yang Xiang, Ming Ji

**Affiliations:** 1 Department of Physiology, Xiangya School of Basic Medical Sciences, Central South University, Changsha 410078, China; 236511006@csu.edu.cn (R.Z.); zhangweiran0418@163.com (W.Z.); quxiangping@csu.edu.cn (X.Q.); xiangyang@csu.edu.cn (Y.X.); 2 Key Laboratory of General University of Hunan Province, Basic and Clinic Research in Major Respiratory Disease, Changsha 410078, China; 3 National Experimental Teaching Demonstration Center for Medical Function, Changsha 410013, China

**Keywords:** 8-oxoguanine DNA glycosylase 1, inflammation, age-related disorders, tumors, DNA damage repair

## Abstract

Inflammation, malignant tumors, and age-related disorders are all associated with oxidative DNA damage. 8-oxoguanine DNA glycosylase 1 (OGG1), which recognizes and repairs intracellular oxidative damage, was initially thought to play a pivotal role in cellular repair of such damage. However, a growing body of evidence now indicates that OGG1 not only participates in DNA oxidative damage repair but also possesses transcription factor activity, closely linked to the development and progression of oxidative DNA damage-related diseases. We propose that OGG1 can repair damaged DNA, while in certain diseases, OGG1 promotes transcription and exacerbates disease progression. This review discusses the mechanisms of action of OGG1 and proposes it as an emerging therapeutic target for curing the aforementioned diseases.

## 1. Introduction

Reactive oxygen species (ROS) are defined as oxygen radicals, including superoxide anion radicals (O_2_^−^) and hydroxyl radicals (OH), as well as radical-derived non-radical oxidants, such as hydrogen peroxide (H_2_O_2_) and singlet oxygen (^1^O_2_) [1]. When intracellular levels of ROS exceed the processing capacity of the scavenging enzyme system, a state of oxidative stress ensues. In a state of oxidative stress, excess intracellular ROS attack DNA, leading to oxidative DNA damage (including DNA base oxidation, double- and single-strand breaks, intra- and inter-strand cross-links, and the formation of an apurinic/apyrimidinic site (AP site). This damage is an important factor in triggering inflammation, malignancy, and age-related diseases [2,3].

It is evident that among the four DNA bases, guanine (G) possesses the lowest oxidation potential and is the most susceptible to oxidation, resulting in the formation of 8-oxoguanine (8-oxoG) [4]. As the most prevalent form of oxidative DNA damage, 8-oxoG is produced in substantial quantities and may exert significant deleterious effects. Consequently, 8-oxoG has emerged as a significant biomarker for evaluating oxidative stress and oxidative DNA damage in both animal models and human subjects [5]. OGG1 is the specific recognition element for 8-oxoG [6], thereby initiating the base excision repair (BER) pathway for the remediation of small base lesions [7]. This enables OGG1 to play a role in protecting DNA integrity and regulating cellular health. A growing body of research has identified a significant role for OGG1 in the transcriptional regulation of inflammatory factors. Consequently, OGG1 is regarded as a viable therapeutic target for the treatment of inflammation, cancer, and age-related diseases.

This review systematically synthesizes the impact and mechanism of action of OGG1 in various diseases, and explores new ideas and therapeutic targets for the study of the pathogenesis of some clinical diseases.

## 2. The Structure and Function of the Mammalian DNA Glycosylase Family: OGG1

In mammalian cells, five DNA glycosylases have been identified as specific recognition elements for oxidizing bases. These enzymes are classified into two distinct families: the Nth family, also known as the Endonuclease III-like Family, and the Nei family, which is designated as the Endonuclease VIII-like Family. The nomenclature of these families is derived from the endonuclease III (EcNth) and endonuclease VIII (EcNei) of Escherichia coli, respectively. The Nth family consists of OGG1 and NTH1 (Endonuclease III-like Protein 1), while the Nei family is composed of NEIL1 (Nei-like DNA Glycosylase 1), NEIL2, and NEIL3 [8].

The gene encoding OGG1 in the human genome was cloned and characterized in a number of laboratories [9,10,11,12,13,14]. The gene that encodes OGG1 is located at locus 25 on the short arm of chromosome 3 (chromosome 3p25) and consists of eight exons that give rise to seven variable splicing forms. Depending on the last exon, *OGG1* can be classified into two types: α-subtypes (with exon 7, 1a and 1b), and β-subtypes (with exon 8, 2a–2e). Of these, type 1a and type 2a mRNAs are predominantly expressed in human tissues. Type 1a mRNA is transcribed from exons 1 (the promoter) to 7 and encodes a protein of 345 amino acids (39 kDA) (α-OGG1), which is localized to the nucleus and mitochondria. In contrast, type 2a mRNA is transcribed from exons 1 to 6 plus exon 8, resulting in a protein of 424 amino acids (47 kDA) (β-OGG1), which is localized exclusively in mitochondria [15]. The involvement of β-OGG1 in the repair of 8-oxoG damage in mitochondria has been demonstrated; however, the role of α-OGG1 in the repair of mtDNA (mitochondrial DNA) damage remains to be elucidated [16].

### 2.1. Base Excision Repair Function of OGG1

**Figure 1:**
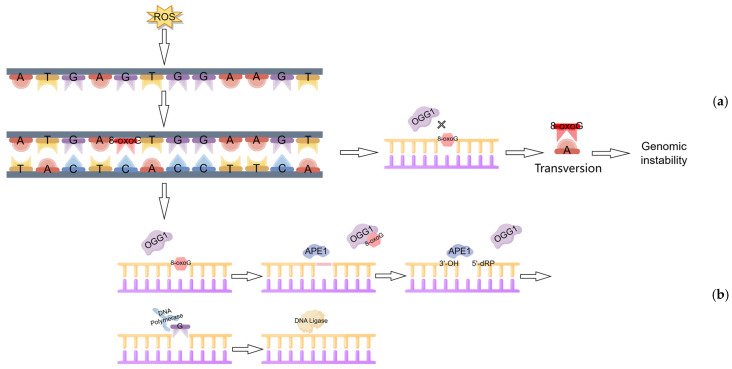
DNA repair mechanism and BER pathway of OGG1: (**a**) Oxidative stress can lead to G:C to T:A transversion mutations leading to genomic instability. (**b**) OGG1-BER pathway diagram. (Created with Figdraw).

During DNA replication, 8-oxoG is prone to being misread as thymine (T) by DNA polymerase, resulting in a preferential mismatch with adenine (A). This process leads to a G:C to T:A transversion mutation, which contributes to genomic instability [17]. OGG1 specifically recognizes 8-oxoG through its glycosylase activity, forming a stable complex via hydrogen bonding and hydrophobic interactions. It hydrolyzes the N-glycosidic bond between the damaged base and deoxyribose, releasing 8-oxoG from the DNA strand to generate an apurinic (AP) site. Through its β-lyase activity, OGG1 cleaves the phosphodiester bond at the 3′-end of the AP site, producing a 3′-phospho-α,β-unsaturated aldehyde terminus (3′-PUA). Subsequently, endonucleases (e.g., APE1) excise the aldehyde moiety of 3′-PUA via their 3′-phosphodiesterase activity, yielding a 3′-OH end. However, under physiological conditions, OGG1’s β-lyase activity is markedly inefficient. Consequently, the majority of AP sites are directly cleaved at the 5′-end by APE1, generating 3′-OH and 5′-deoxyribose phosphate (5′-dRP). A DNA polymerase then incorporates the correct nucleotide, and DNA ligase seals the nick to restore an intact DNA strand, ultimately achieving complete restoration of the original DNA sequence [18,19]. As illustrated above, the BER pathway is initiated by OGG1, and it is the primary mechanism for repairing specific guanine oxidative lesions induced repairing oxidative DNA damage by ROS [20] (Figure 1).

OGG1’s primary function is the repair of DNA damage, a process that contributes to the maintenance of cellular health and the subsequent functioning of tissues and organs. This, in turn, has the potential to reduce the risk of some diseases.

A growing body of research has revealed that OGG1 plays a multifaceted role in cellular processes, including DNA damage repair and non-DNA damage repair enzyme activities. For instance, OGG1 has been observed to regulate the expression of inflammation-related genes, thereby contributing to the development of inflammatory conditions [21].

### 2.2. OGG1 Can Function as a Transcription Factor

The researchers found that during interphase, hOGG1 is localized to the nuclear matrix and sparse chromatin, while during mitosis, hOGG1 follows the condensed chromatin and is phosphorylated on serine residues in vivo. Subsequent studies revealed that both purified and nuclear matrix-associated hOGG1 were substrates for protein kinase C (PKC)-mediated phosphorylation in vitro, thereby providing the first evidence of the post-translational modification of hOGG1 in organisms [22].

In order to investigate the role of hOGG1 in gene expression, researchers stimulated mouse lungs with 8-oxoG via nasal drops. RNA sequencing (RNA-Seq) analysis was then performed to assess the changes in the whole transcriptome. The results of this analysis showed that out of 23,337 transcripts identified, 8-oxoG induced up-regulation of 983 transcripts and down-regulation of 1398 transcripts. The up-regulated transcripts were found to be associated with a variety of biological processes, including homeostasis, the immune system, macrophage activation, regulation of fluid surface tension, and response to stimuli. In particular, they were closely related to homeostatic and immune system processes [23].

#### 2.2.1. Binding of hOGG1 to Endogenous 8-oxoG Leads to Pro-Inflammatory Gene Expression

Endogenous 8-oxoG is produced by the attack of intracellular ROS on the C8 position of guanine (G) in DNA. Boldogh, I. et al. further demonstrated that OGG1 exhibits a high degree of binding affinity for endogenous 8-oxoG at non-substrate sites, a specific region in enzymes or other biological macromolecules that does not directly bind substrates but regulates biological functions by interacting with effector molecules, thereby forming the OGG1-8-oxoG complex. This complex interacts with Ras, a member of the typical superfamily of small GTP-binding proteins, by facilitating the exchange of guanine nucleotides. This, in turn, increases the GTP-bound form of Ras and initiates signaling for transcriptional activation of downstream genes [24]. Concurrently, Hajas, G. et al. ascertained that in the presence of 8-oxoG, OGG1 interacts with the GDP-binding Rac1 protein, thereby facilitating the conversion of GDP to GTP. Furthermore, they determined that an elevated intracellular level of 8-oxoG corresponds to an increased proportion of GTP-binding Rac1 protein. Subsequent to this, Rac1-GTP traverses the nuclear membrane-associated type IV NADPH oxidase, culminating in an augmentation of ROS. This finding further validates that the OGG1-8-oxoG complex has GEF (guanine-nucleotide exchange factor) activity [25].

However, a clear link between the OGG1-8-oxoG complex and cell signaling has not been rigorously established. In this vein, German, P. et al. built upon this foundation by exploring the link between OGG1-BER and cell signaling using KG-1 cells (human acute myeloid leukemia cells) expressing the temperature-sensitive mutant OGG1. It was found that activated Ras phosphorylates downstream Raf1, mitogen-activated protein kinase (MAPK) kinases MEK1,2, and extracellular signal-regulated kinases ERK1,2 [26]. Subsequently, Aguilera-Aguirre, L. et al. posited that the OGG1-8-oxoG complex has the capacity to displace GDP bound to Kirsten rat sarcoma viral oncogene homolog (K-RAS), Neuroblastoma RAS viral oncogene homolog (N-RAS), and Harvey-RAS (H-RAS), replacing it with GTP, thereby inducing activation of KRAS, MAPK, PI3K, MSK, and NF-κB signaling pathways. This, in turn, has been shown to lead to pro-inflammatory gene expression and inflammatory cell accumulation [27]. Furthermore, Luo, J. et al. discovered that OGG1 can physically interact with RhoA. In the presence of 8-oxoG, OGG1 activated the RhoA GTPase, which mediated the polymerization of α-smooth muscle actin (α-SMA) into stress fibers and increased the levels of α-SMA in insoluble cell/tissue fractions. This verified the link between oxidative stress and altered cellular structure [28].

The aforementioned results indicate that the OGG1-8-oxoG complex has guanine nucleotide exchange factor activity and is able to activate small GTP-binding proteins.

#### 2.2.2. OGG1 Binds to Exogenous 8-oxoG to Inhibit Inflammation

**Figure 2:**
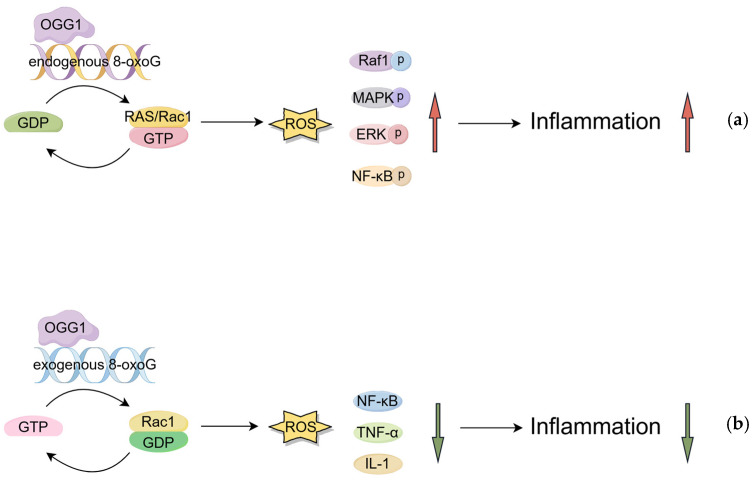
Non-repair function of OGG1 and regulation of inflammatory signaling pathways: (**a**) OGG1 binds to endogenous 8-oxoG to promote inflammation. (**b**) OGG1 binds to exogenous 8-oxoG to inhibit inflammation. (Created with Figdraw).

Ock, C. et al. found that exogenous 8-oxoG has the opposite function to endogenous 8-oxoG. Exogenous 8-oxoG is generated either indirectly through environmental factor-induced ROS or by direct DNA damage from such factors. In a mouse model of acute inflammation, exogenous 8-oxoG was found to inhibit the activation of Rac1, a member of the small GTPase family, and to attenuate the NF-κB signaling pathway, thereby acting as an anti-inflammatory agent. To test this hypothesis, researchers established several animal models of acute inflammation. Intraperitoneal injection of LPS into mice induced severe lung tissue inflammation, whereas exogenous administration of 8-oxoG treatment reduced the expression of TNF-α as well as pro-inflammatory mediators interleukin (IL)-1, IL-6, IL-18, cyclo-oxygenase-2, and inducible nitric oxide synthase [29], and this inflammation-reducing efficacy was even superior to the effect of aspirin [30]. In the context of an OVA-induced asthma mouse model, the investigation revealed that the experimental group exhibited a reduction in inflammatory cell infiltration, diminished collagen deposition and protein expression, and decreased expression of matrix metalloproteinase (MMP)-2/-9, in comparison to the control asthma mice that were not administered exogenous 8-oxoG treatment. Furthermore, it has been demonstrated to impede the expression of a variety of inflammatory mediator proteins, including IL-4, IL-5, IL-8, IL-13, TNF-α, and IFN-γ, and to diminish STAT1 and NF-κB activities [31]. Other researchers have proposed that exogenous 8-oxoG may function as a regulatory molecule in the context of oxidative stress-induced gastritis, operating through mechanisms such as antagonizing Rac-GTP binding or impeding signals that contribute to the progression of gastritis [32] (Figure 2). The potential of synthetic exogenous 8-oxoG as a therapeutic or preventative agent for gastrointestinal inflammation warrants further investigation.

#### 2.2.3. The OGG1-8-oxoG Complex Can Act as a Transcriptional Coactivator to Promote Gene Expression

In addition to the OGG1-8-oxoG complex activating/antagonizing small GTP-binding proteins, OGG1 was found to interact with 8-oxoG in the promoter region of target genes and act as a transcriptional co-activator to promote gene expression [33]. In the presence of tumor necrosis factor-α (TNF-α)-induced ROS, there is an increase in the level of 8-oxoG within the promoter region of the genome. This increase subsequently leads to the recruitment of OGG1 to the promoter region of the chemokine ligand 2 (CXCL2) molecular target, which is present in both mice and humans. OGG1 has been shown to enhance *cxcl2* expression by promoting the recruitment of several key factors, including transcription initiation factor II-D (TFIID), NF-κB/RelA, Sp1 (specificity protein 1, which functions mainly in the active center of RNA polymerase), and phosphorylated RNA polymerase II (p-RNA Pol II). In a mouse model of airway inflammation, OGG1 deletion has been shown to reduce the binding of transcription factors to the promoter, thereby inhibiting *cxcl2* expression and leading to the attenuation of TNF-α-mediated innate immune responses (IIRs) [18,34]. The aforementioned results suggest that OGG1 plays an important role in the transcriptional activation of pro-inflammatory genes under oxidative stress conditions.

### 2.3. OGG1 Induces DNA Demethylation

Oxidative stress has been conclusively proven to exert a significant impact on DNA methylation levels [35]. The TET (Ten-Eleven Translocation) protein family serves as the core enzymes in mammals that execute active DNA demethylation, initiating this process through the oxidation of 5-methylcytosine (5mC) [36]. It has been elucidated that OGG1 functions as a signaling molecule in oxidative stress-induced DNA demethylation. The authors established the interaction between OGG1 and TET1 through co-immunoprecipitation (Co-IP) assays, demonstrating that OGG1 could pull down TET1 protein. Then, a robust correlation has been identified between the repair of 8-oxoG and the demethylation of methylated cytosine-phosphate-guanine (CpG) islands. Oxidative stress has been demonstrated to induce the recruitment of the OGG1/TET1 complex to the 8-oxoG site. The process under investigation has been shown to promote the conversion of neighboring 5mC to 5-hydroxymethylcytosine (5hmC), 5-formylcytosine (5fC), and 5-carboxycytosine (5caC). This conversion process ultimately results in the reversion of these modifications back to normal cytosine [37]. Methylation/demethylation has been demonstrated to modify genes at the transcriptional level, thereby affecting the level of translational expression of the gene. Methylation is commonly linked to gene silencing, particularly in promoter regions, which can impede the binding of transcription factors to DNA and diminish gene expression levels. OGG1-induced DNA demethylation suggests a potential involvement in gene expression regulation.

### 2.4. OGG1 and Histone Arginine Modification

Histone arginine modification is regarded as a critical post-translational modification that contributes to the formation of chromatin and the regulation of transcription [38,39]. OGG1 plays a pivotal role in this process. In their study, Wang, W. et al. examined the impact of OGG1 on the asymmetric dimethylation of histone H4 at arginine 3 (Arg3) (H4R3me2a) under conditions of oxidative stress. Immunofluorescence assays revealed that OGG1 translocates into the nucleus under oxidative stress. Subsequently, Co-IP experiments demonstrated that OGG1 interacts with protein arginine methyltransferase 1 (PRMT1). This interaction enhances asymmetric dimethylation of histone H4 at arginine 3 (H4R3me2a), promoting chromatin relaxation and recruiting the transcription factor YY1 (Yin Yang 1). Consequently, these changes facilitate transcriptional activation of the *c-Myc* gene [40].

The above results suggest that OGG1 possesses not only a base excision repair function but also a role in gene transcription.

## 3. Role of OGG1 in the Development of Inflammation

Inflammation is defined as a series of complex reactions involving the body’s immune system, including the clearance of pathogens and the repair of damaged areas. The lungs are the site of gas exchange between the human body and the external environment; therefore, the lungs are constantly subjected to various environmental pollutants, toxins, and allergens from the outside world [41]. When an inflammatory response occurs in the lungs, many inflammatory cells are activated and release cytokines and mediators to coordinate the process of inflammation development. This is essential to maintain the homeostasis of the lungs and to ensure normal lung function. Research has demonstrated a close relationship between the development of inflammation and the presence of ROS and 8-oxoG. OGG1, on the one hand, has been shown to initiate BER repair of oxidative DNA damage, and on the other hand, to play a role in the inflammatory response by affecting the functions of various immune cells [42]. For instance, when OGG1 is defective, mice exhibit resistance to acute and systemic inflammation [43]. The objective of this study is to examine the potential impact and role of OGG1 in the development and progression of inflammatory diseases.

### 3.1. OGG1 Promotes Inflammatory Responses in Asthma

Asthma is characterized by chronic airway inflammation [44]. ROS are signaling molecules that trigger respiratory inflammation, exacerbating asthma symptoms by stimulating smooth muscle contraction, histamine release from mast cells, mucus production, and inducing pro-inflammatory gene expression [45,46].

The study constructed a mouse model of asthma induced by ovalbumin (OVA), which showed inflammatory cell infiltration in the lungs of the model mice, with a significant increase in the levels of pro-inflammatory cytokines IL-4, IL-13, IL-8, and TNF-α, as well as an increase in the level of 8-oxoG in the BALF (bronchoalveolar lavage fluid). These findings suggest that oxidative DNA damage is involved in the development of asthma. It has been posited that the development of asthma is associated with oxidative DNA damage [47]. This hypothesis was proposed by Tanner, L. et al. An evaluation was conducted of the role of TH5487, a small molecule inhibitor of OGG1, in an OVA-induced asthma model. It was found that administration of TH5487 to OVA-induced asthma mice significantly inhibited the proliferation of goblet cells and the production of mucus. As indicated by the results of this study, there was a substantial decrease in mouse plasma IgE levels and the recruitment of eosinophils and other immune cells into the lungs. Furthermore, the expression of IL-4, IL-5, and IL-13 in BALF was also significantly reduced. In addition, airway hyperresponsiveness induced by OVA stimulation was significantly attenuated [48]. The authors attribute this phenomenon to TH5487 inhibiting OGG1-DNA binding at 8-oxoG-enriched promoter sites. However, a significant limitation remains the absence of detailed mechanistic exploration, such as structural analyses or binding affinity assays. The aforementioned results demonstrate the clinical relevance of OGG1 as a pharmacological target for the treatment of allergic asthma.

### 3.2. Role of OGG1 in COPD

Chronic obstructive pulmonary disease (COPD) is a heterogeneous chronic inflammatory disease of the airways [49], the pathogenesis of which is not yet fully understood. Deslee, G. et al. have demonstrated that the lungs of patients suffering from severe COPD exhibit a high degree of DNA oxidative damage. RT-PCR detection of OGG1 expression in human lung tissues reveals a significant increase in OGG1 mRNA expression in the lungs of patients with very severe COPD compared to those with mild, moderate, severe, and normal healthy lung tissues. This has been suggested as a marker of oxidative stress in the lungs [50].

In a recent study, researchers treated peripheral blood mononuclear cells (PBMCs) isolated from COPD patients and healthy subjects (smokers and non-smokers) with UFPs (ultrafine particles). The results indicated that UFPs induced an increase in the expression of OGG1 mRNA in PBMCs from Acute Exacerbation of COPD (AECOPD) patients, leading to the release of IL-18 and IL-33. However, this phenomenon was not observed in PBMCs from patients with stable COPD. This phenomenon is associated with mitochondrial dysfunction and an increase in reactive ROS production in patients with AECOPD. The expression of NLRP3, as well as mitochondria-derived ROS (mtROS), was found to be significantly higher in COPD patients than in healthy subjects when the level of oxidative stress was examined in PBMCs. Furthermore, a significant increase in the content of 8-oxoG was observed. Notably, UFP stimulation has been observed to induce a marked increase in the mRNA expression level of OGG1 in PBMCs from non-smokers. In contrast, a decrease in OGG1 expression has been noted in PBMCs from smokers, while no significant difference has been detected in PBMCs from AECOPD patients [51]. This phenomenon may be attributed to the release of oxidized mtDNA (8-oxo-dG) during the process of programmed cell death, which has been observed to bind to NLRP3 and thereby inhibit the activation of inflammatory vesicles [52].

As indicated by the literature, an imbalance of the oxidative–antioxidant system constitutes a significant pathogenic factor in the development of COPD [53]. A body of experimental evidence suggests a link between a polymorphism in exon 7 of the OGG1 gene and a serine–cysteine (Ser326Cys) exchange at codon 326. This exchange has been shown to affect oxidative stress-related life activities, including a substantial reduction in DNA damage repair [54]. Evidence from both animal models and human studies indicates that XRCC1 can coordinate and stimulate OGG1 activity [55]. This collaborative relationship between XRCC1 and OGG1 is crucial in the development of COPD, suggesting that the *XRCC1* gene plays a pivotal role in the BER pathway [56].

Therefore, we hypothesize that increased expression of OGG1 may serve to alleviate COPD.

### 3.3. OGG1 Has Different Effects on Lung Fibrosis Induced by Different Factors

Pulmonary fibrosis is a progressive disease accompanied by inflammatory damage and destruction of tissue structure [57]. Transforming growth factor beta (TGF-β), central to the pathogenesis of pulmonary fibrosis, promotes extracellular matrix accumulation [58]. Persistently activated TGF-β1 signaling mediates the transition of lung fibroblasts and epithelial cells, driving fibrosis progression [59]. Researchers ascertained that OGG1 interacted with the TGF-β/Smad axis to promote the progression of pulmonary fibrosis in a BLM (bleomycin)-induced mouse model of pulmonary fibrosis. This was further validated by the finding that *Ogg1* deficiency attenuated bleomycin-induced pulmonary fibrosis by constructing *Ogg1^−/−^* mice [60]. Concurrently, Pan, L. et al. discovered that 8-oxoG levels were augmented in the accessible chromatin of mouse lungs following TGFβ1-induced injury. They further ascertained that the binding of OGG1 to 8-oxoG led to the recruitment of transcription factors, including phospho-SMAD3, to pro-fibrotic gene promoters, thereby facilitating gene activation [61].

A recent study reported that TH5487 blocked the binding of OGG1 to 8-oxoG-containing DNA substrates, resulting in reduced immune cell recruitment and alleviation of inflammation in mouse lungs [43]. Song, C. et al. found that the expression level of OGG1 increased during the activation phase of lung fibroblasts in the BLM-induced mouse lung fibrosis model. OGG1 has been shown to promote the expression of fibroblast activation markers through its interaction with Snail1. This process is dependent on the recognition and nuclear localization of 8-oxoG, and it is independent of lytic enzyme activity and mitochondrial localization. Furthermore, the use of TH5487, a pharmacological inhibitor of OGG1, in the middle stage of lung fibrosis, has been shown to alleviate BLM-induced lung fibrosis in murine models [62]. Ling, H. et al. found that [63], the polyubiquitination and subsequent degradation of OGG1, induced by TH5487, was facilitated by the E3 ubiquitin ligase NEDD4L. This process led to the inhibition of the alveolar epithelial–mesenchymal transition (EMT) process and the deposition of the extracellular matrix (ECM) in the lungs.

As established by Tanner, L. et al. [64], TH5487 exhibited efficacy in reducing pro-inflammatory mediator levels, inflammatory cell infiltration, and lung remodeling in a male C57BL/6J mouse model of lung fibrosis induced by bleomycin. TH5487 was also assayed for its capacity to reduce pro-inflammatory mediator levels, inflammatory cell infiltration, and lung remodeling levels. Concurrently, the analysis of lung tissue samples from IPF patients indicated that the interaction of OGG1 and mothers against decapentaplegic homolog 7 (SMAD7) induced fibroblast proliferation and differentiation. The above studies suggest that OGG1 promotes the development of inflammation in pulmonary fibrosis and is a potential molecular target for the treatment of pulmonary fibrosis.

However, in a mouse model of PM2.5-induced pulmonary fibrosis, the level of oxidative DNA damage in the lungs of mice was elevated when they were exposed to particulate matter for a period of time, and OGG1 deficiency exacerbated PM2.5-induced oxidative stress, death of type 2 alveolar epithelial cells (AEC2s), and pulmonary fibrosis in mice [65]. A subsequent study revealed that silent information regulator sirtuin 3 (SIRT3) overexpression led to the restoration of OGG1 levels and activity, the prevention of mtDNA damage, and the suppression of downstream inflammatory gene overactivation. Additionally, SIRT3 overexpression rescued PM2.5-induced lung fibroblast-to-myofibroblast transition (FMT) [66]. We propose that in PM2.5-induced pulmonary fibrosis, OGG1 primarily repairs damaged DNA to mitigate the disease, operating through distinct mechanisms compared to its role in BLM-induced pulmonary fibrosis.

The study’s findings indicate that OGG1 may exert variable effects on distinct etiologies of pulmonary fibrosis, with its role being particularly pronounced in the mitigation of particulate matter-induced pulmonary fibrosis.

### 3.4. OGG1 and Atherosclerosis

Atherosclerosis is a chronic inflammatory lesion that occurs in the intima of arteries [67], and increased oxidative stress is a major feature of hypercholesterolemia-induced atherosclerosis. A substantial accumulation of 8-oxoG has been identified in atherosclerotic plaques induced by a high-cholesterol diet [68]. Immunohistochemistry revealed that 8-oxoG was predominantly present in the superficial layers of plaques containing substantial numbers of macrophage-derived foam cells. Furthermore, 8-oxoG exhibited a strong positive signal in all cell types of the plaque, including macrophages, VSMC (vascular smooth muscle cells), and endothelial cells [69].

OGG1, a pivotal enzyme, plays a critical role in the repair of 8-oxoG in human vascular smooth muscle cells (hVSMCs). Its acetylation serves as a regulatory mechanism for protein stability, thereby influencing the repair function of OGG1. The researchers ascertained that in hVSMCs with atherosclerotic plaques, impaired repair of 8-oxoG in the nucleus was associated with reduced OGG1 acetylation. p300/sirtuin 1 is a major regulator of OGG1 acetylation/deacetylation, and both proteins are able to interact with OGG1 to regulate OGG1 activity at the endogenous level and thereby reduce oxidative damage-induced plaque formation due to oxidative damage. However, p300 levels were found to be down-regulated in human plaque VSMCs and in response to oxidative stress. This suggests that the ROS-induced reduction in OGG1 acetylation may be associated with decreased p300 expression [70].

Animal experiments have also demonstrated a role for OGG1 in the development of atherosclerosis. NLRP3 inflammasome-mediated activation of IL-1β secretion has become an important component of the inflammatory process in atherosclerosis. Tumurkhuu, G. et al. utilized a low-density lipoprotein receptor (LDLR) knockout mouse, as well as an *Ogg1^−/−^Ldlr^−/−^* mouse model. The results demonstrated that in atherosclerotic lesions, mtDNA oxidation, inflammatory vesicle activation, and apoptosis were increased in *Ogg1^−/−^Ldlr^−/−^* mice, and serum IL-1β and IL-18 secretion was higher than in *Ldlr^−/−^* mice. Bone marrow transplantation from *Ogg1^−/−^* mice into *Ldlr^−/−^* mice resulted in more severe atherosclerotic lesions and increased IL-1β production. It was also found that microRNA-33 (miRNA-33), a molecule associated with atherosclerosis, can directly inhibit the expression of human OGG1 and indirectly inhibit the expression of mouse and human OGG1 through AMP-dependent protein kinase. OGG1 deficiency has been shown to cause a significant increase in oxidized mitochondrial DNA (mtDNA), which, in turn, has been linked to the activation of the NLRP3 inflammasome, the secretion of interleukin-1 beta (IL-1β), and the acceleration of atherosclerosis. In contrast, in normal mice, OGG1 plays a protective role in atherogenesis by preventing excessive activation of NLRP3 inflammatory vesicles [71]. A related study has shown that OGG1 may inhibit lipid accumulation in plaques through non-NLRP3 inflammatory vesicle-related pathways, which in turn affects the atherosclerotic process. A mounting body of evidence suggests a potential for OGG1 to play a role in the treatment of atherosclerosis, thus offering a novel target for clinical therapeutic interventions [72].

Concurrently, the mechanism of action of OGG1 in atherosclerosis necessitates further study.

### 3.5. Down-Regulation of OGG1 Expression Exacerbates Gastrointestinal Inflammation

*H. pylori* is a bacterium that is present in the human gastric mucosa and has been linked to various gastrointestinal diseases, including chronic gastric sinusitis, duodenal ulcer, gastric atrophy, and gastric adenocarcinoma [73] Prolonged infection of *H. pylori* can result in the release of ROS and reactive nitrogen species (RNS) from inflammatory cells, which can lead to oxidative damage to DNA and the production of 8-oxoG. Research revealed that during *H. pylori* infection, OGG1 expression is up-regulated to remove 8-oxoG lesions. However, due to ROS levels far exceeding the physiological range, persistent OGG1 activation generates excessive AP sites. Downstream APE1, potentially limited by insufficient expression or impaired activity, fails to promptly process the accumulated AP sites. This leads to AP site accumulation in DNA duplexes and increased double-strand breaks (DSBs) in gastric epithelial cells. Subsequent studies revealed that the silencing of OGG1 using RNA interference (RNAi) led to a protective effect against the accumulation of excessive AP sites within the cells [74]. This finding indicates that *H. pylori*-induced oxidative damage and the initiation of the BER pathway in normal gastric epithelial cells may result in genomic instability.

Not coincidentally, some researchers have observed an effect of OGG1 on intestinal inflammation and the composition of gut microbes [75,76]. A comprehensive analysis of the gut microbial composition of *Ogg1^−/−^* mice fed diverse diets revealed substantial alterations in the gut microflora of mice in the control and high-calorie diet groups. The increased microbes were found to be associated with energy intake. Furthermore, the increase in pro-inflammatory microorganisms in *Ogg1^−/−^* mice rendered them more sensitive to dextran sulfate sodium (DSS)-induced acute enteritis [76]. The expression levels of DNA repair proteins OGG1, XPA, MLH1, PARP1, and XRCC6 in ulcerative colitis and sporadic colorectal cancer tissues were examined using immunohistochemistry. The results demonstrated that all the DNA repair proteins were expressed at higher levels in comparison to normal colonic mucosa [77]. These results suggest that the repression of OGG1 expression in intestinal inflammation may amplify the body’s inflammatory response.

## 4. The Role of OGG1 in Immune Senescence

Aging is frequently accompanied by immune system dys-regulation, which can result in increased circulating pro-inflammatory cytokines [78]. In this process, low-level chronic inflammation is referred to as “inflammatory aging” [79,80,81]. Inflammatory aging (Inflammageing) is a sterile, low-grade chronic inflammation that progressively increases with age. This phenomenon is not merely indicative of advanced age; it can also precipitate health deterioration, physical debilitation, and the onset of age-related diseases.

In the context of ongoing “inflammatory aging,” the brain exhibits a pronounced inflammatory response in many age-related neurological disorders [82]. It has been established that DNA undergoes damage as the aging process unfolds. The genomic instability that ensues from the accumulation of DNA damage has been linked to DNA mutations and cognitive impairment [83,84].

### 4.1. Mitochondrial OGG1 Alleviates Aging-Induced Neuroinflammation in Mice

Research revealed a decline in the expression of OGG1 [85] and the activity of mitochondrial OGG1 [86] in the mouse brain with advancing age. Consequently, to investigate the function of mitochondrial OGG1 in age-related neuroinflammation, researchers developed a model of naturally aging mitochondria-targeted mtOGG1^Tg^ mice, which exhibited high levels of human OGG1 expression. The results demonstrated that mitochondrial expression of OGG1 modulated the activation of downstream pro-inflammatory factors TNFα, IL1β, and IL6 by inhibiting the expression of pSTING, thereby attenuating hippocampal inflammation in male mice. Furthermore, the researchers detected the expression of 8-oxoG in mouse brain tissues by immunofluorescence and observed the brain tissue structure and mitochondrial length using electron microscopy. They found that mtOGG1 reduced the accumulation of 8-oxoG in the brain tissues of male mice. These results suggest that elevated mitochondrial OGG1 expression contributes to a reduction in age-related neuroinflammation in male mice [87].

### 4.2. OGG1 and Age-Related Neurodegenerative Diseases

Aging is the most significant factor in the development of neurodegenerative diseases [88]. The investigation revealed that both patients with Alzheimer’s disease (AD) and Alzheimer’s disease model mice exhibit higher levels of 8-oxoG accumulation and DNA damage compared to their respective control groups [89]. A subset of patients exhibits deleterious mutations in *OGG1*, resulting in reduced or absent protein activity. Furthermore, AD patients carrying *OGG1* mutations demonstrate significantly higher levels of 8-oxoG lesions compared to AD patients without *OGG1* mutations [90]. These results suggest that OGG1 may serve as a potential target for the treatment of AD. Jacob, K.D. et al. investigated two mutation types of OGG1, A53T and A288V, and showed that the two mutation types were defective in their catalytic activity. This defect affects the cell’s ability to repair oxidatively damaged 8-oxoG in DNA. The decreased ability of A53T protein to bind DNA substrates and the decreased activity of A288V β-lyase may be the cause of this defect [91]. The above findings indicate that reduced OGG1 activity contributes to AD pathogenesis.

Not coincidentally, Pao, P. et al. evaluated the role of class I histone deacetylase (HDAC1) in the aging mouse brain and Alzheimer’s disease, and found that HDAC1 could interact with OGG1 and enhance the activity of OGG1 in the aging brain. A team of researchers constructed brain-specific knockout mice of *Hdac1* and subsequently performed a comet assay on the hippocampal homogenates. These samples were then subjected to a comet assay to detect the extent of DNA damage and immunofluorescence to detect the expression of 8-oxoG. The researchers found that the knockout mice showed significantly more DNA damage and increased accumulation of 8-oxoG in the brain compared to the control group [92]. These results suggest that reduced OGG1 activity may exacerbate brain damage in AD patients.

Parkinson’s disease (PD) is the second most prevalent age-related neurodegenerative disease [93,94]. A growing body of research has indicated that there is an elevated level of mitochondrial 8-oxo-dG (a nucleotide for 8-oxoG) expression in PD nigrostriatal neurons [95]. This finding suggests a potential relationship between OGG1 and the development of PD.

We therefore propose that the accumulation of 8-oxoG, driven by OGG1 mutations or attenuated OGG1 activity, constitutes a pathogenic mechanism underlying these disorders.

## 5. OGG1 Attenuates Cerebral Ischemia–Reperfusion Injury and Exacerbates Renal Ischemia–Reperfusion Injury

Oxidative stress is closely linked to ischemia–reperfusion injury (IRI) [96]. In cerebral ischemia–reperfusion (CIR) models, multiple antioxidants exhibit neuroprotective properties [97]. A study reported that the OGG1 peptide and its activity exhibited a significant increase following 90 min of cerebral ischemia and 20–30 min of reperfusion in murine models. Furthermore, the augmentation in brain OGG1 protein levels and activity demonstrated a positive correlation with the escalation of DNA damage induced in brain indicator genes (*c-fos* genes) subsequent to forebrain ischemia and reperfusion. The researchers thus concluded that the up-regulation of OGG1 expression reduces oxidative gene damage in the brain, thereby attenuating forebrain ischemia–reperfusion injury [98].

**Figure 3:**
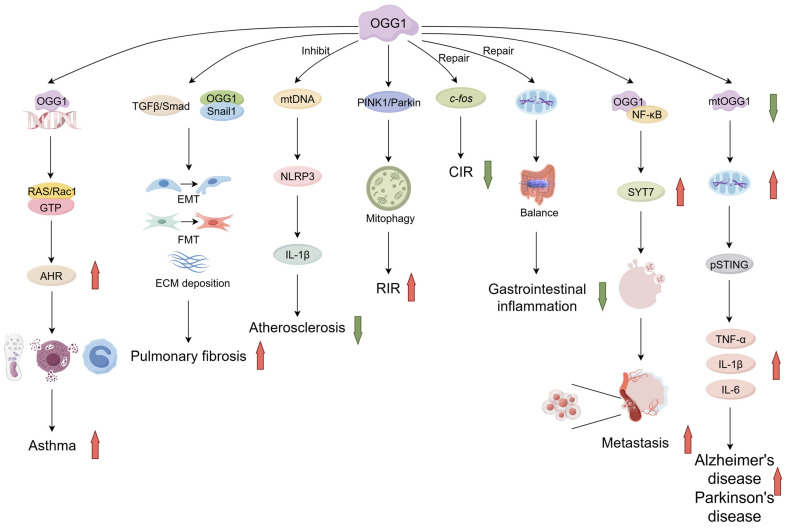
Dual role of OGG1 in disease with differences in molecular mechanisms. OGG1 promotes asthma, pulmonary fibrosis, renal ischemia–reperfusion, Alzheimer’s disease, Parkinson’s disease, and tumor metastasis, and inhibits atherosclerosis, cerebral ischemia–reperfusion, and gastrointestinal inflammation. (Created with Figdraw).

Renal ischemia–reperfusion injury (RIRI) is typically initiated when perfusion is restored following a transient reduction in or cessation of blood flow to the organ. During the process of ischemia–reperfusion, injured cells secrete pro-inflammatory factors and chemokines. Although reperfusion restores aerobic metabolism in the kidneys, it simultaneously generates ROS, causing damage to cells [99]. A comprehensive analysis of the GEO database was conducted by researchers, revealing that following renal ischemia–reperfusion, there was an up-regulation in the expression of *Ogg1*, *Pink1*, and *Bnip3*. KEGG enrichment analysis unveiled substantial disparities in the biological processes of mitochondrial autophagy and base excision repair. Furthermore, the outcomes of TUNEL and HE staining experiments substantiated that renal ischemia–reperfusion injury exhibited a reduced severity following OGG1 knockdown, in comparison to the control group. A similar conclusion was reached in the use of the OGG1 small molecule inhibitor TH5487. Subsequent studies revealed that OGG1 could interact with PINK1 and down-regulate the expression of PINK1, suggesting that OGG1 may negatively regulate mitochondrial autophagy by modulating the PINK1/Parkin pathway, thereby exacerbating renal ischemia–reperfusion injury [100] (Figure 3).

## 6. Role of OGG1 in Tumor Immunity

It was ascertained that OGG1 is localized to chromosome 3p25, a region that exhibits frequent loss of heterozygosity (LOH) in lung and kidney tumors [101]. Subsequently, Audebert, M. et al. conducted an analysis of alterations in the OGG1 gene in 99 cases of renal tumors, identifying several polymorphisms and the occurrence of abnormally spliced OGG1 transcripts [102].

Non-small cell lung cancers (NSCLCs) represent the most prevalent form of lung cancer [103]. Platinum-based drugs represent the prevailing treatment option for patients with advanced inoperable NSCLC. Platinum-based drugs are a primary treatment option for patients with advanced inoperable NSCLC. Polymorphisms in DNA BER genes can affect DNA repair capacity and may alter sensitivity to platinum-based chemotherapy. A body of research has indicated that *OGG1* Ser326Cys, *XRCC1* Arg399Gln, *APE1* Asp148Glu, and *APE1*-141T/G polymorphisms may serve as useful predictors of clinical outcomes in patients with advanced inoperable NSCLC who will receive platinum-based chemotherapy [104]. Singh, A. et al. have also demonstrated that *OGG1* and *MUTYH* gene polymorphisms have an impact on overall survival in lung cancer patients treated with platinum-based chemotherapy [105].

Another study found that *Ogg1^−/−^Myh^−/−^* mice had reduced survival after exposure to the carcinogen benzo[a]pyrene compared to wild-type mice [106].

The aforementioned studies suggest the importance of oxidative stress, oxidative DNA damage repair, and characterizing the role of gene polymorphisms in cancer.

Ma, Y. et al. constructed a model of low-level oxidative stress promoting lung cancer cell metastasis, which was used to investigate how OGG1 affects extracellular vesicle (EVs) release and thus cancer metastasis. A study was conducted that revealed a specific interaction between OGG1 and 8-oxoG in the human lung adenocarcinoma cell line A549. This interaction was found to occur within the transcription factor NF-κB, which plays a crucial role in regulating gene expression. The study found that OGG1 binds to NF-κB, facilitating its binding to the promoter region of the activating synaptic binding protein 7 (SYT7). This binding event subsequently promotes the transcription of SYT7, leading to the release of EVs and, ultimately, facilitating tumor metastasis. The use of Th5487, a substrate activity binding inhibitor of OGG1, blocked the recognition and delivery of oxidative signals, reduced SYT7 expression, and inhibited EV release. In contrast, the use of its β-lyase activity inhibitor, OGG1-IN-08, was found to be not required for its enzyme cleavage activity to function in transcriptional regulation [107].

The integrity of mtDNA plays an important role in breast cancer progression and metastasis. Therefore, Yuzefovych, L.V. et al. constructed mice lacking OGG1 (KO), mice overexpressing the human OGG1 subunit 1α in mitochondria (Tg), and mice simultaneously lacking OGG1 and overexpressing the human OGG1 subunit 1α in mitochondria (KO/Tg). The results indicated that tumors in Tg and KO/Tg mice were significantly smaller than those in KO and wild-type (WT) mice. Additionally, there were fewer metastatic foci and a lower rate of metastasis [108].

## 7. OGG1 Small-Molecule Drugs: Limitations and Outlooks

Bioaffinity drugs are defined as medications that modulate target function through specific intermolecular interactions [109]. OGG1 small-molecule inhibitors and activators such as TH5487, SU0268, and TH10785 are bioaffinity pharmaceuticals. TH5487 and SU0268 act by specifically binding to the OGG1 enzyme, thereby blocking its ability to recognize and repair 8-oxoG, while also interfering with its non-repair transcriptional regulatory functions [110]. TH10785 forms hydrogen bonds and hydrophobic interactions with Phe319 and Gly42 in the active site pocket of OGG1, thereby altering the enzyme’s conformation and significantly enhancing its catalytic efficiency [111]. Currently, TH5487 remains in preclinical development and has not entered clinical use [112], while SU0268’s development was discontinued due to its off-target inhibition of the ABCB1/MDR1 drug efflux pump. TH10785, designed for DNA repair in AD, may increase DNA double-strand breaks and potentially induce genomic instability. Thus, developing novel bioaffinity pharmaceuticals targeting OGG1 is imperative.

## 8. Summary

The significance of OGG1 in preserving the equilibrium of the immune system, as an indispensable component of the initiation of the BER pathway, is of paramount importance. Numerous studies have demonstrated that OGG1 plays a pivotal role in inflammation, cancer, and age-related diseases. These diseases are frequently accompanied by inflammation, and OGG1 exhibits a dual role, demonstrating notable variations in its function during inflammation of disparate tissues and organs due to diverse etiologies. These variations may be attributed to the presence of distinct OGG1 phenotypes within different diseases and tissues and organs. For instance, in the context of gastrointestinal inflammation and atherosclerosis, OGG1 exhibited a capacity to impede the progression of inflammation, a phenomenon that can be attributed to the predominance of β isoforms over α isoforms. Conversely, in the bleomycin-induced pulmonary fibrosis model, OGG1 demonstrated a pro-inflammatory function, a property that can be explained by the prevalence of α isoforms. The present study of OGG1 is chiefly centered on its function in oxidative damage. Nevertheless, OGG1 also possesses non-DNA damage repair enzyme functions that merit further exploration. Researchers have developed small-molecule inhibitors TH5487, SU0268, and activator TH10785 for OGG1 [113,114]. The significant research potential and clinical value of OGG1 justify a thorough investigation.

## Abbreviations

OH	hydroxyl
^1^O_2_	singlet oxygen
5caC	5-carboxycytosine
5fC	5-formylcytosine
5hmC	5-hydroxymethylcytosine
5mC	5-methylcytosine
8-oxoG	8-oxoguanine
AD	Alzheimer’s disease
AECOPD	Acute Exacerbation of COPD
AHR	airway hyper-reactivity
AP	apurinic/apyrimidinic
BALF	bronchoalveolar lavage fluid
BER	base excision repair
BLM	bleomycin
CpG	cytosine-phosphate-guanine
CIR	cerebral ischemia–reperfusion
COPD	chronic obstructive pulmonary disease
CXCL2	C-X-C motif chemokine ligand 2
DSBs	double-strand breaks
ECM	extracellular matrix
EMT	epithelial–mesenchymal transition
FMT	fibroblast-to-myofibroblast transition
GEF	guanine nucleotide exchange factor
H_2_O_2_	hydrogen peroxide
HDAC1	class I histone deacetylase
H-RAS	Harvey-RAS
IPF	idiopathic pulmonary fibrosis
K-RAS	Kirsten rat sarcoma viral oncogene homolog
Ldlr	low-density lipoprotein receptor
mtDNA	mitochondrial DNA
mtROS	mitochondrial ROS
N-RAS	Neuroblastoma RAS viral oncogene homolog
NSCLCs	non-small cell lung cancers
O_2_^−^	superoxide anion
OGG1	8-oxoguanine DNA glycosylase 1
OVA	ovalbumin
PBMC	peripheral blood mononuclear cell
PD	Parkinson’s disease
PF	pulmonary fibrosis
PKC	protein kinase C
redox	reduction–oxidation
RIR	renal ischemia–reperfusion
RNA-Seq	RNA sequencing
ROS	reactive oxygen species
SIRT3	silent information regulator Sirtuin 3
SMAD	drosophila mothers against decapentaplegic protein 7
SSBs	single-strand breaks
SYT7	synaptic binding protein 7
UFPs	ultrafine particles
VSMCs	vascular smooth muscle cells
α-SMA	α-smooth muscle actin
